# Knowledge-building in an environment mediated by digital technology: A case study in higher education

**DOI:** 10.1007/s10639-022-11304-0

**Published:** 2022-09-14

**Authors:** Judith Martín-Lucas, Ángel García del Dujo

**Affiliations:** Department of Theory and History of Education, Faculty of Education, University of Salamanca, Salamanca, Spain

**Keywords:** Education, Technology, Re-ontologising, Higher order thinking, Learning theory, Higher education

## Abstract

The advancement of technology in recent years seems to be prompting a re-ontologising of the world. Digital technology is transforming the educational spaces we inhabit, as well as our way of processing information. Although there are already numerous studies that have addressed this technological reality, only a handful have done so from a theoretical perspective. That is why we present research that seeks to reinforce the latest theoretical contributions for understanding how modern technology may be affecting the way in which knowledge is built. Based on the latest research in social constructivism, this is a qualitative study designed to contribute to the creation of a specific theoretical framework for an onlife world. An ill-structured task and a semi-structured interview were used to observe the use of the thinking skills that enable us to build knowledge and the relationship between them. The results show that the ways of building knowledge are changing, as digital technology fosters the use of higher-order thinking skills that, furthermore, operate in a chaotic, complex, and unpredictable manner. In conclusion, this study upholds the notion that the ways of building knowledge are changing, but we still need more empirical contributions to create a generally accepted theoretical construct for explaining how we build knowledge through digital technology.

## Introduction

We live in a society in which few things remain untouched by technology, whether they are objects, actions or behaviours, either because of its appeal and its way of making everyday tasks easier, or simply because it is hard to ignore. Our society is therefore the most technological one that has ever existed. Our modern technology and the hyperconnectivity associated with it are altering our way of processing information. A technology that is steadily becoming more autonomous, while at the same time increasing and expanding our own cognitive abilities (Heersmink, 2015). In an ever-increasing manner, we are assigning technology a significant number of our everyday tasks, thereby enabling us to satisfy a large part of our needs through a simple click.

Technology has always been a part of human history and progress, yet the nature and scale of its development in recent years has led some scholars to contend that our world is undergoing a process of re-ontologising (Floridi, 2014), with significant changes and transformations in all spheres of life. These transformations are also being reflected in education; the autonomy, versatility, and ubiquity that new devices provide are changing all the rules of the learning game, beginning with the very scenarios in which teaching takes place. The ease with which we shift from one scenario to another is now referred to as education in onlife environments*.*

Although the science of education has focused on issues related to this phenomenon in recent years, in most cases it has done so without resorting to theory (Sánchez-Rojo & Martín-Lucas, 2021), concerning itself mainly with addressing new methodologies and their effects on academic performance (Hew et al., 2019). Research in education has shown more interest in studying technology from a didactic and instrumental perspective, dealing more with what that technology can offer us as “means to an end”, without stopping to think that, regardless of the use we make of it, this technology also educates, as it shapes minds (García del Dujo et al., 2021). The research into this technology does not appear to find an answer to the way in which new digital artifacts affect our way of learning, our way of building knowledge.

Faced with this approach, we agree with Hew and et al. (2019) that the future of education also depends on the development of a language and theoretical framework that enable us to understand how this technology is (re)formulating our ways of learning and building knowledge. Therein lies the purpose of this article: to present research that seeks to reinforce the latest theoretical contributions for understanding how modern technology may be affecting the way in which knowledge is built.

## Toward a theory of education in an onlife world

Although it is fair to say that several attempts have been made to explain how learning takes place in virtual environments, and whether or not this technology is changing our way of learning, the studies conducted over the past ten years do not appear to be sufficiently robust to constitute a theoretical framework for identifying how knowledge is built within a scenario permeated by digital technology (García del Dujo & Martín-Lucas, 2020; García del Dujo et al., 2021). Nevertheless, the latest trends agree that an updated theory of learning should at least tackle two issues: on the one hand, the potential that technology has to release us from certain mental tasks (Pettersson, 2021), and on the other, this same technology’s ability to migrate learning spaces to the digital environment (Liu & Zhang, 2022), and even create new ones. Hence, the reason we consider that the framework for interpreting learning that is consistent with the times we are living in should be informed by the following two aspects: first, the legacy of the theories of social constructivism; secondly, the understanding of technology as a facilitator of higher-order thinking processes.

### The legacy of social constructivism

Many of the approaches adopted in the twentieth and twenty-first centuries are rooted in the pedagogical corpus of social constructivism, and more specifically in its activity theory, developed over three generations (Engeström, 2001; Leont’ev, 1978; Vygotsky, 1979). This theory is defined mainly by considering the mediating component of social, historical, and cultural artifacts within the learning process; in other words, contextualising the individual, accepting that interpersonal precedes intrapersonal, wherein learning becomes a process of auto-social knowledge-building, and all cognitive processes are influenced by the context and its prevailing culture. It is specifically on this approach that today’s theoretical studies associated with this construct focus their attention: on the role that digital technology plays as a mediating and contextual artifact in learning and knowledge-building processes. This has given rise to other theoretical approaches along these lines, such as situated learning theory (Lave & Wenger, 1991) and distributed cognition theory (Salomon, 1993), which have been used to study the digital environment as a contextual feature that generates new spaces in learning processes (Rivoltella, 2014).

These steps toward the consideration of learning as a situated and distributed action have prompted the emergence of two of the trends arising from Vygotsky’s thesis, namely, distributed cognition theory (Hutchins, 1995) and extended mind theory (Clark, 1996), which focus on the brain-artifact combination, and enable us to understand how we use artifacts and tools in the learning process. These approaches have been extended over the past decades by Kirsh (2006), Sutton (2006), and Heersmink (2017), Heersmink and Knight (2018); their main contribution focuses on attributing a cognitive *status* to modern technology, depending on whether this technology manages to integrate deeply or superficially according to the artifacts’ level of coupling with mental processes.

Other theories, such as connectivism (Siemens, 2005), have stressed the hyperconnectivity of digital technology, whereby learning arises through the ability to link informational nodes in a chaotic manner. This latter approach has been severely questioned (Harasim, 2017; Kop & Hill, 2008; Ovalles, 2014; Zapata-Ros, 2015); nevertheless, we consider that, from an educational perspective, this approach provides valuable contributions that guide a theoretical construct that enables us to clarify whether the digital environment could be altering the ways of building knowledge. Accordingly, over the past decade some scholars have contended that technology’s cognitive *status*, and its ability to release us from having to undertake certain tasks (Floridi, 2014), could foster the use of higher-order thinking skills (DeSchryver, 2009, 2017; Loh & Kanai, 2016).

### Higher-order thinking in the new theory of learning

**Fig. 1:**
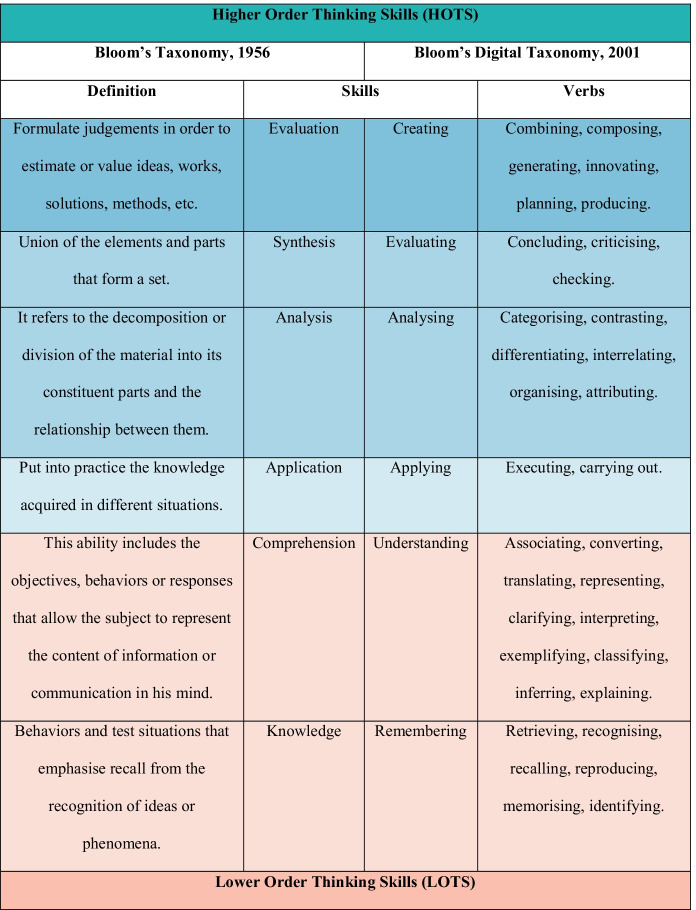
Evolution of Bloom’s taxonomy

Higher-order thinking skills play a key role in today’s learning processes because they support learning-to-learn (Behnagh & Yasrebi, 2020; Lu et al., 2021). The concept of higher-order thinking, nonetheless is not new, as its origins are to be found in Newmann (1990) and Lipman (1991). According to these authors, skills such as memorisation pertain to lower levels of thinking, while others, such as interpretation and analysis are associated with higher-order thinking. Although there is no consensus on the definition of this term, the literature does agree that higher-order thinking involves the undertaking of mental processes that enable us to make complex inferences that entail the full integration of the new information into existing knowledge (Afflerbach et al., 2015). From an educational perspective, it may be affirmed that higher-order thinking processes require the deployment of a series of cognitive skills that call for greater complexity and self-regulation, and therefore a higher cognitive effort than for those considered to be of a lower order (Johansson, 2020; Lee & Choi, 2017). There are different studies and theories on this matter that have sought to enrich and operationalise the concept of higher-order thinking by classifying lower-order thinking skills into a higher level. The contribution that has so far received the widest acceptance is Bloom’s taxonomy (Bloom, 1956) and its application to the digital environment, giving rise to Bloom’s digital taxonomy (Anderson & Krathwohl, 2001). Both taxonomies consider that skills are arranged in a hierarchical manner; in other words, achieving a more complex skill necessarily requires completing a simpler skill (see Fig. 1).

One of these taxonomies’ main advantages is that they provide scholars with an excellent tool for measuring and understanding how knowledge is built. Hence, the reason that even the most recent research and theoretical approaches, such as web-mediated knowledge synthesis theory, are based on taxonomies of this nature.

### The theory of web-mediated knowledge synthesis

**Fig. 2:**
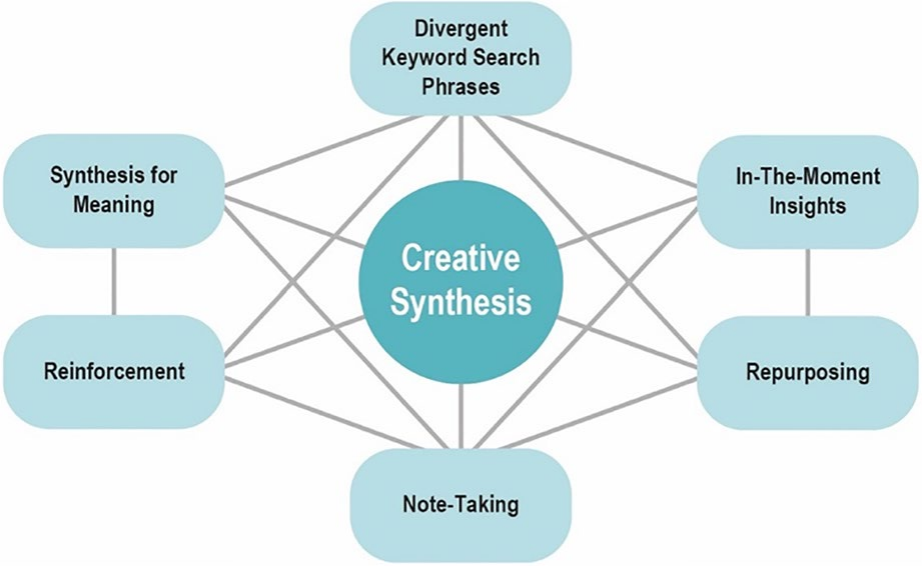
Theory of web mediated knowledge synthesis

The *Theory of Web-Mediated Knowledge Synthesis* was propounded in 2012 by Michael DeSchryver. This scholar posits that the use of modern technology has meant that both learning and knowledge-building are being undertaken in a chaotic manner, given the overabundance of multiformat data, and the versatility, ubiquity and speed provided by such technologies as the internet. According to DeSchryver (2012, 2014), our way of building knowledge through digital technology involves two types of synthesis: the synthesis of meaning, understood as the development of the comprehension of implicit and explicit meanings in the content being addressed, and generative synthesis, as a creative act through which an individual creates, designs, and builds knowledge. Generative synthesis is detected through seven skills (see Fig. 2) that, according to DeSchryver, may be addressed individually and simultaneously, and interrelated with other different skills in the knowledge-building process.

According to this theory, the most superficial levels of synthesis are related to the synthesis of meaning (convergent search words, repurpose, reinforce), while the deeper levels are closely linked to creative synthesis (note-taking, in-the-moment insights, divergent search words). In view of its nature, the web fosters the use of creative or generative cognitive skills – closely related to higher-order thinking skills. This approach even leaves the way open for the possibility that knowledge may often emerge incidentally, thanks to the versatility and myriad possibilities that the internet provides.

The theory of web-mediated knowledge synthesis is so far the only theoretical approach to social constructivism that has a sound grounding of an empirical nature, although it is still very limited. DeSchryver himself admits that his contribution requires empirical support to allow creating a new medium on which to visualise learning in digital times, spaces and technologies. Hence, the reason we are backing the DeSchryver approach through this study, in which our aim, from a socio-constructivist perspective, is to analyse, explore and understand whether the use of digital technology and the connectivity it provides have an influence on the way we build knowledge.

## Research questions and research aims

This study sets out to further the discussion and theorisation on how knowledge is built in environments mediated by digital technology. Specifically, the following questions are addressed:

- Are there any differences in the use of strategies and procedures for building knowledge with and without the support of digital technology?
- How are the thinking skills involved in knowledge-building related?

## Methods

This is a qualitative study (Mittenfelner Carl & Ravitch, 2016; Trigueros et al., 2018), conducted during the academic years 2019/2020 and 2020/2021. Specifically, the research design involves a case study (Stake, 2000), with the aim being to conduct a detailed analysis of the use and experience of digital technology in knowledge-building. Although part of the study was conducted during the COVID-19 pandemic, this did not affect its technical development, as the experiments proceeded with complete normality, while at all times abiding by socio-health recommendations.

### Sample

Convenience sampling was used (seeking the participation of different knowledge branches), involving thirteen students aged 21 to 25, in the third year of their degree courses in the five branches of knowledge; Humanities: Degree in English Studies; Social Sciences: Degree in Educational Science; Health Sciences: Degree in Medicine; Engineering: Degree in Computer Engineering, and Sciences: Degree in Biology.

The sample consisted of eight women (62%) and five men (38%).

### Instruments

The design of the research called for instruments for observing the type and relationship of the thinking skills students use when building knowledge, as well as place the design of these instruments within the context of socio-constructivist educational research. This has involved the use of two instruments: an ill-structured task (Collins et al., 2016; Jonassen, 1997; Laxman, 2010) and a semi-structured interview. The very nature of ill-structured tasks means that the results of a learning activity depend on thinking skills conditioned by the environment and the context within which they take place (Bixler & Land, 2010; Collins et al., 2016; Ouyang et al., 2021). Moreover, these kinds of tasks do not have a single solution, thereby requiring the people tackling them to reason, reflect and use different kinds of thinking skills until they find one, which means producing a large amount of quality data.

**Fig. 3:**
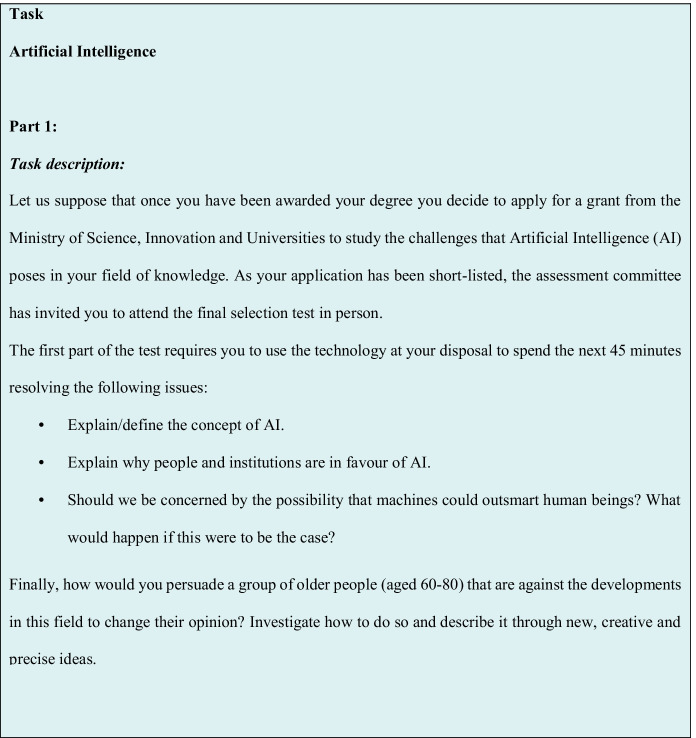

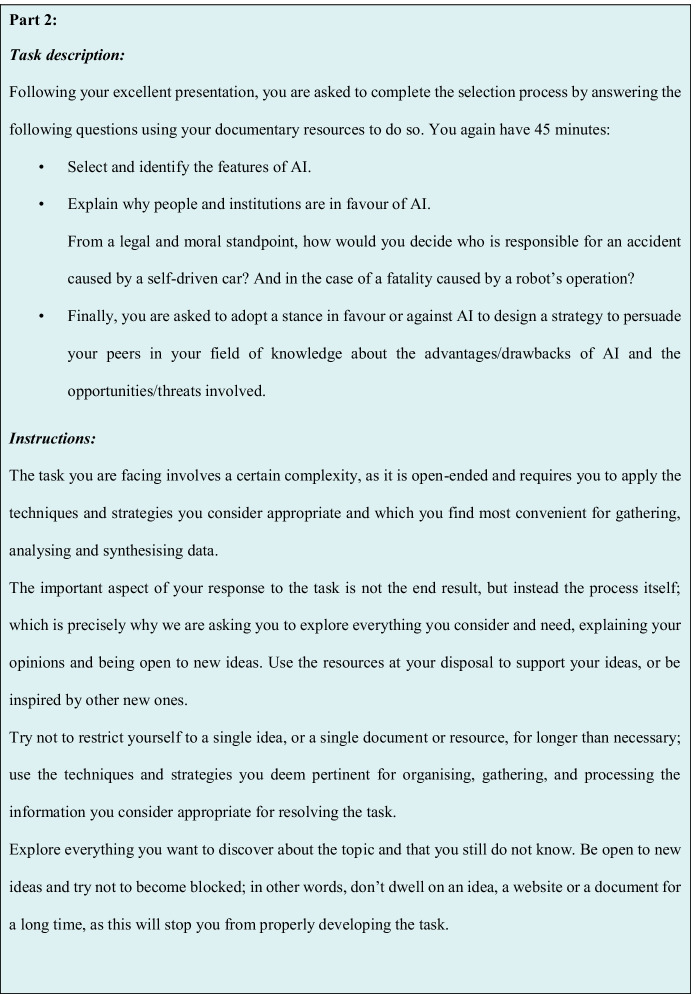
Ill-structured task

The ill-structured task (see Fig. 3) used here was designed according to the criteria agreed by a number of experts in the application of these kinds of problems (Collins et al., 2016; Jonassen, 2000; Laxman, 2010), whereby we contextualised the problem in the form of a current issue. Furthermore, the task was divided into four parts. The first one involved contextualising and generally considering the problem in order to situate the individual in a specific time and place; the second one involved fostering the use of skills related to the synthesis of meaning and lower-order thinking skills. The third part consisted of fostering the use of skills related to creative and generative skills and higher-order thinking skills, and finally, a moral dilemma was presented. The task’s design was validated by a panel of experts and its application in a pilot study (Martín Lucas, 2020, 2021). Three different task proposals were drawn up, with two different topics (AI and paediatric oncology), which met the requirements for their consideration as an ill-structured task. Following their evaluation, the experts unanimously agreed on the proposal that involved AI, as the most suitable topic for this research.

The semi-structured interview was used to increase the information obtained in the ill-structured task. Given that the pilot study detected a lack of information in certain aspects that were important for the research, this interview was designed to extend the information on three dimensions of “judgement and validation” (Patton, 2015) and seven subdimensions: task development (strategy and difficulties), use of technology (convenience), and use of thinking skills (memory, insights, treatment and processing of information, and creative synthesis).

### Data collection procedure

The experiment was held in a Gesell chamber at Salamanca University’s Faculty of Education. This type of facility is used to allow researchers to observe and listen to participants while they are resolving the ill-structured task.

The participants were required to complete both parts of the ill-structured task; the first one with the backing of digital technology, in which the students were permitted to use their laptop, smartphone or tablet to go online and use their apps. The first part of the task involved using a word processor to respond. The second part did not require the use of digital technology resources, which meant that the students in this case could use only printed texts and books. Furthermore, they used pen and paper to complete the ill-structured task. Based on the researcher’s observations and the script that had been drafted and validated beforehand, the semi-structured interview was then held. The average time taken to resolve the ill-structured task was one hour and fifteen minutes, and each interview lasted 20 min on average.

The data were collected by recording the individual’s spoken thoughts, the notes made during the task, the compilation and storage of the browser history when digital technology was used, and the collection of printed texts and books when not using digital equipment. All the data were recorded in audio and video format and then transcribed with a view to analysing the information through **NVivo v.12** and Gephi 0.9.2 software, according to a system of deductive categories.

### Data analysis

The data gathered from the ill-structured task were analysed via a categorical approach (Packer, 2017). A map of categories was drawn up (see Table 1), based on the theoretical paradigms and instruments presented in preceding sections. This map was designed to illustrate the convergence between three theoretical aspects: the theory of web-mediated knowledge synthesis, the prior theories of social constructivism, and the categorisation of thinking skills (learning taxonomies). This categorisation was validated by a panel of experts and its application in the trial test (Martín Lucas, 2021a).

**Table 1:** Category map of the study

Remembering	Recovering information from the long-term memory	Identify	Locate knowledge in the long-term memory
		Recoup	Recover specific information from the long-term memory
Search strategies	Series of search operations and problems for resolving a problem or task	Divergent search words	One or more words that are not explicitly found in the task instructions
		Convergent search words	All the search words that are explicitly found in the task description
		Plan	Set out a way of undertaking a task
Synthesis of Meaning	Combining, reorganising, rewriting, deducing and summarising internet data that lead to an understanding of the implicit or explicit meaning of the texts found	Combine	Unify data from different sources
		Reorganise	Re-establish and/or relocate the information at our disposal
		Rewrite	Express words and ideas in a different way
		Deduce	Draw conclusions on the idea/information being addressed
		Summarise	Reduce the content, the information, to briefer and more concise terms
		Understand	Find links between prior and new information through the interpretation, exemplification or explanation of the content being used
Analysing	Breaking the material down into its constituent parts and determining how these parts are related to each other and to the overall structure	Differentiate	Screen, select, and distinguish between relevant and irrelevant information
		Organise	Find coherence, integrate, highlight or structure the way in which the parts of a message are arranged and structured
		Attribute	Deconstruct, describe and identify the message’s purpose
		Evaluate	Reach conclusions according to standardised criteria or based on established norms or patterns
In-the-moment insights	Intuitions, ideas that arise in the moment and need not necessarily be directly linked to the general task or to the reason for browsing on the web, although they are related to the current learning context	A resource	The insight emerges from a resource
		Multiple resources	The insight emerges from several resources
		An unconnected activity	The insight emerges from a resource or information retrieved from memory that does not involve the resource being accessed
		A combination of the two previous ones	In-the-moment insight or intuition from a combination of the resource being used and an unconnected activity
Repurposing	Modify existing ideas in a substantial and generative way through their reformulation, merger or other forms of combination, while retaining one or more important qualities from the original idea	Redo	Do again; reform; reformulate
		Edit	Modify or adapt a text
		Reuse	Use a resource again
		Qualify	Fine-tune, delicately graduate; express differences
Reinforcement	This occurs when a multiplicity of resources facilitates the strengthening of an idea when finding it in a similar way to other resources	Insights	Reinforcement arises through previous insights
		Explicit	It emerges through explicit content in the resource
		Implicit	It emerges through implicit content in the resource
Using	Apply knowledge in specific situations	Resolution	Use knowledge to solve problems or make decisions
		Investigation	Use knowledge to enquire or conduct investigations
Creative Synthesis	Create new meaning. Appearance of new aspects of the synthesis in sequence or jointly, with the outcome being the formulation of a solution to the problem in hand. It emerges through the exploration of resources and individual perceptions. It constitutes a generative way of interacting with the resource (analogue or digital), prior knowledge, notes, and the description of the task	Originality	Reveal unprecedented innovation
		Independence	Reject standard alternatives
		Imagination	Suggest aspects of possible worlds
		Holistic	Understand meanings of the parts in terms of the whole
		Divergent	Discordant, discrepant
		Connection de ideas	Unify, introduce or link two concepts, opinions or points of view
		Generate	Rearrange items into a new pattern or structure

The data from the semi-structured interviews were analysed through three dimensions: Task development (strategy and difficulties), Use of technology (convenience), and Use of thinking skills (memory, insights, data treatment and processing, and creative synthesis). An inductive categorisation process was used (Mittenfelner Carl & Ravitch, 2016). This process enabled us to draw up a map of categories for analysing the data gathered from the interviews.

## Results

### Ill-structured task

**Fig. 4:**
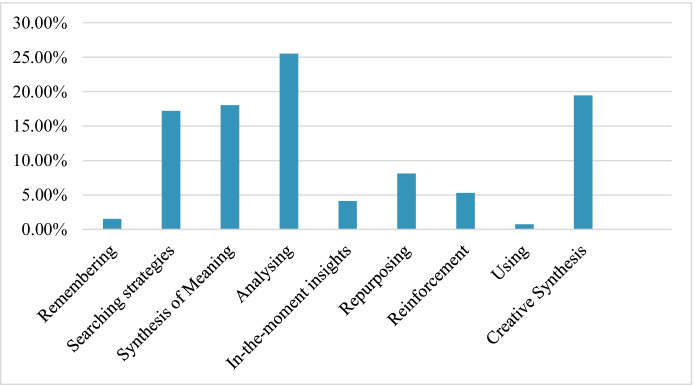
Main categories used according to the percentage of units of analysis coded

The results show that the categories recording a lower percentage of coverage were *Use* (0.76%) and *Remembering* (1.51%). By contrast, the categories that recorded a higher percentage of coverage were *Analyse* (25.51%) and *Creative synthesis* (19.46%), followed by *Synthesis of meaning* (18.05%) and *Search strategies* (17.19%) (see Fig. 4).

**Fig. 5:**
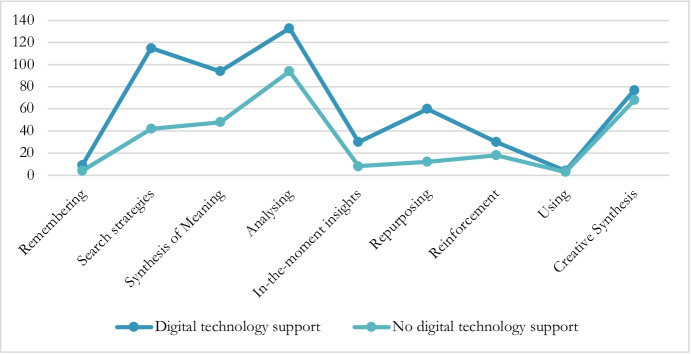
Rate of use of categories depending on the type of technology. Note. The figure shows the rate at which the categories are used by the participants in the study depending on whether or not they resolved the task with the support of digital technology

The next step involved analysing the relationship between the use of digital technology and the various thinking skills. Figure 5 shows how the participants, when carrying out the task with the support of digital technology, recorded a higher rate of use for all the skills except for *Use*.

**Fig. 6:**
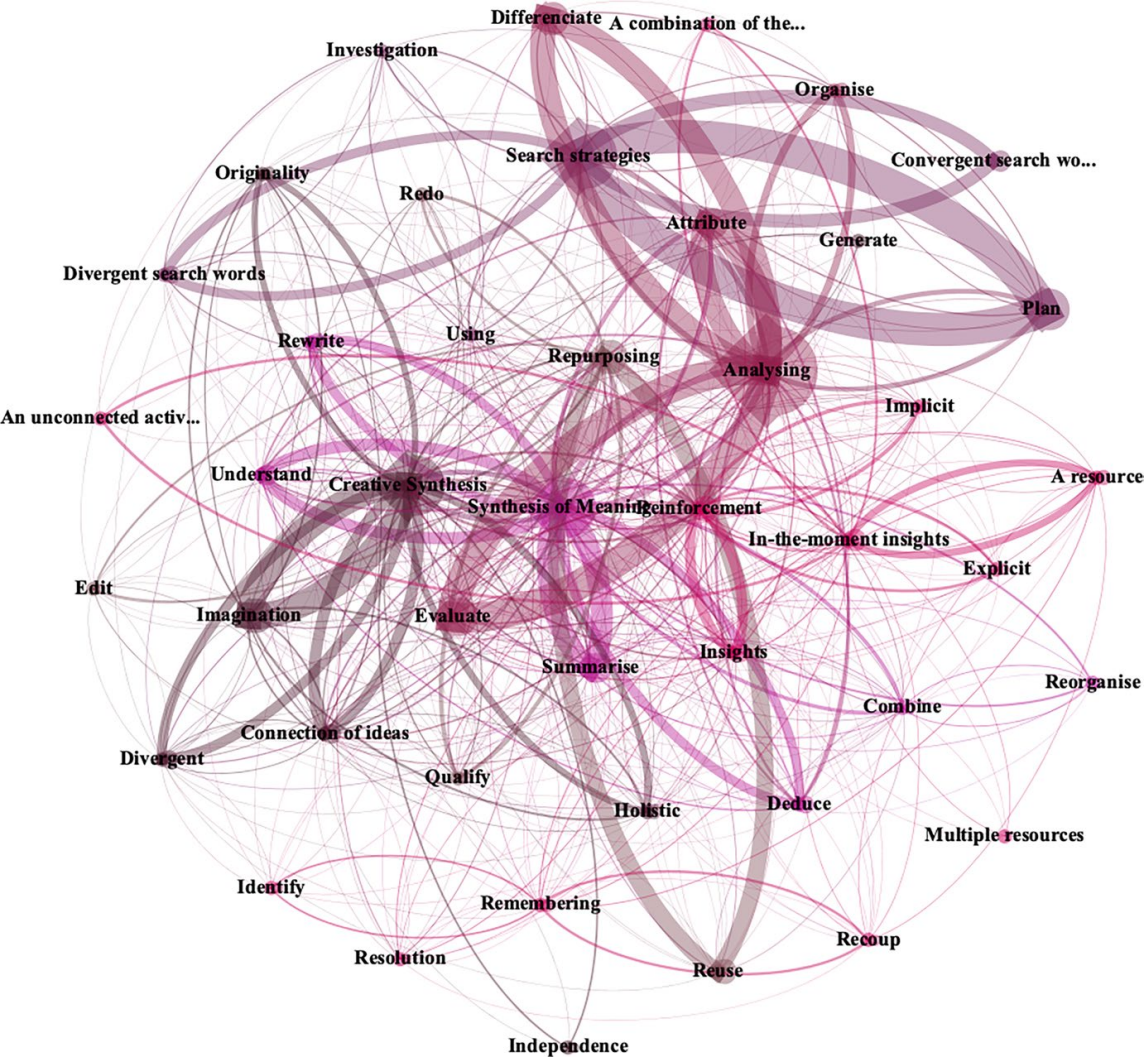
Graph of the relationships between nodes and sub-nodes

A study was subsequently conducted on the relationship between categories and subcategories; this involved obtaining the matrix of nodes and sub-nodes through the **NVivo v.12** program. The matrix was exported to the Gephi v.0.9.2 program. The data were processed via the application and calibration of the mean degree and degree of modularity, with the application also of the Fruchterman Reingold distribution. Figure 6 shows the nodes that accumulated a higher coding – the larger the node, the higher its percentage of use and coding – and their relationship with all the other nodes and sub-nodes – the thicker the pairs of nodes, the closer their relationship.

**Fig. 7:**
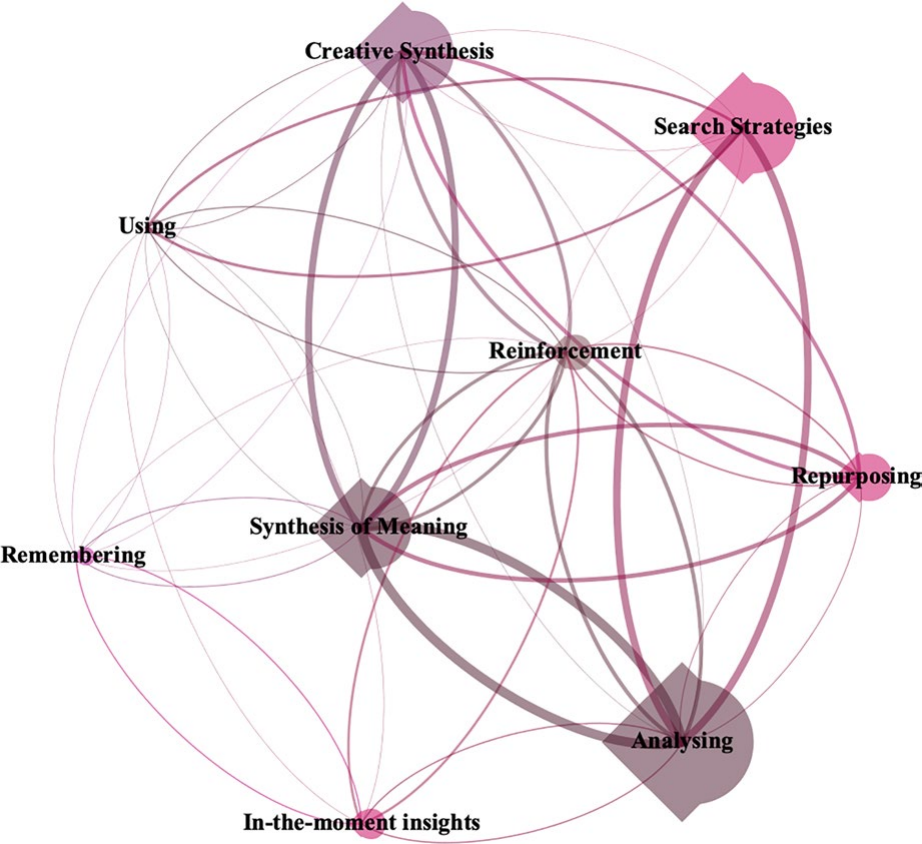
Graph of the relationships between nodes

This figure reveals how all the nodes and sub-nodes in the graph are interrelated. Generally, the graph shows how the categories *Analyse, Search strategies,* and *Creative synthesis* were the ones used the most. Given that the mesh of relationships shown in the graph was highly complex and chaotic, a detailed representation (Fig. 7) was used to show the relationships between the main nodes.

Figure 7 shows how the most recurrent relationships occur between the following pairs of nodes: *Search strategies – Analyse; Analyse – Synthesis of meaning,* and *Synthesis of meaning – Creative synthesis.* In turn, albeit with less intensity, it reflects the relationship between *In-the-moment insights* and *Analyse* and *Remembering,* as well as the relationship between *Search strategies* and *Use.*

### Semi-structured interview

The semi-structured interview provided data of considerable interest that allowed corroborating some of the results forthcoming from the task. Firstly, in their discourse the participants said that *in-the-moment insights* emerge more often when using digital technology. The following are some of the participants’ actual words and opinions:

“Yes, I’ve had them with the virtual side, that is, not at all with the analogue source. But I have with the computer” (ID: 45510).

“I’ve had more with the internet because I’ve accessed more sites, so I’ve read more things, and that’s given me more ideas” (ID: 71648).

The participants also said they preferred to use digital technology rather than pre-digital technology, which they found to have many shortcomings. The following are again some of the participants’ actual words and opinions:

“Digital technology is much more convenient, definitely. I’ve been much more relaxed with the time and less pressured, as all the information is far more accessible because I knew I was going to find everything I was looking for, whereas with books I’ve been more at a loss” (ID:45510).

“I normally work with books, although I have them in PDF format…I tend to look for keywords” (ID:12489).

“My opinion of books is that they are admittedly very reliable sources, you don’t need anything else, but it’s up to you to find the idea, locate the idea you’re looking for, and you don’t really know if it’s the right book, and I don’t think that’s the case with the computer” (ID: 70309).

“They save time. The use of technologies, the computer helps me to go much faster. I use it to find the exact data I need, because with books you start off looking for one thing and you don’t find exactly what you want, while with the internet I find what I’m looking for” (ID: 45510).

“On the internet in my case, I think, because I’ve found everything more quickly, I also write faster with the computer… in the end you basically have a very small device that has numerous options, whether they are platforms such as PubMed, for example, or standard Google, you can find something in a second, it takes much longer with a book…There are some things, for example, that have taken me ages to find in books but I think that if I’d used the computer I would have taken half the time I took with the book” (ID: 12489).

## Discussion

In the light of these results, the educational research on learning in environments mediated by digital technology cannot ignore the fact that our ways of building knowledge are changing. Firstly, our results coincide with those reported in prior studies, conducted before and after the pandemic, showing that the use of digital technology for carrying out a task fosters the use of higher-order thinking skills (Coşgun Ögeyik, 2022; DeSchryver, 2017). Specifically, this technology involves the more frequent use of such skills as Analysis, In-the-Moment Insights, and Creative Synthesis. These results enable us to confirm the hypothesis put forward by Michael DeSchryver (2017), namely that digital technology fosters the use of higher-order thinking skills when we use it as a support for resolving a problem or task.

Our results also show that when building knowledge, our thinking skills do not follow a set or hierarchical pattern of behaviour, but instead flow in a random and unpredictable manner. Our results clearly show how thinking skills not only emerge randomly, but also that a higher-order skill may have a direct link to a lower-order one, and vice versa. An example of this are the direct relationships found between such skills as *synthesis of meaning* and *analyse, creative synthesis* and *reuse, use,* and *search strategies.* These results corroborate the study by DeSchryver (2014), whereby the effect of digital technologies, such as the web, is that thinking skills are used simultaneously and randomly, without following a pre-ordained or established order. We also coincide with the ideas propounded by Siemens (2005), who affirms that the new digital scenario is a chaotic environment, converting learning practices into actions that in most cases are unpredictable. These results, furthermore, enable to move away from Bloom’s taxonomy (1956) and Bloom’s digital taxonomy (Anderson & Krathwohl, 2001), given that, as we have seen, thinking skills do not proceed according to a hierarchical pattern. There is no need to pass first through a lower thinking skill in order to subsequently use a higher-order one. By contrast, the deployment of thinking skills when resolving the task follows a complex pattern of behaviour, a chaotic mesh of relationships in which thinking skills are indistinctively related, whether they are of a higher or lower order, and regardless of whether or not we use digital or pre-digital technology. In sum, our ways of learning and building knowledge seem to have adapted to the digital and informational environment. They are no longer organised hierarchically, as used to be the case, but instead they are presented through impulses and in any direction, horizontally, vertically, and even transversally.

Regarding the results forthcoming from the semi-structured interview, they again corroborate the findings reported by DeSchryver (2017); *In-the-Moment Insights* are used more often in the case of digital technology, which may be because its use means not only accessing a larger amount of data and information more quickly, but also that the information is displayed on screens in different formats and in a more dynamic way, which may foster the frequent appearance of these *insights* and affect our decision-making when resolving a problem. Finally, it is important to note that the participants involved here clearly showed a preference for the use of digital technology when resolving the task.

This study therefore corroborates the approach taken by the theory of web-mediated knowledge synthesis. Nevertheless, and according to DeSchryver (2017) and Hew and et al. (2019), with a view to creating a generally accepted theoretical construct for explaining how we build knowledge in an onlife world, we need more empirical contributions to confirm that we are heading in the right direction. We consider that the instruments designed and validated for this research, as well as the map of categories, could serve as a reference and support for future studies on this phenomenon.

Finally, it should be noted that, as mentioned earlier, the research presented in this paper was only partially affected by the COVID-19 pandemic. From March to June 2019, all face-to-face classes were cancelled, being replaced by Emergency Remote Learning (ERL) (Fulford, 2022; Tulaskar & Turunen, 2022). Nevertheless, this situation is not believed to have seriously impacted upon our research outcomes here, as academic activity at Salamanca University had already been highly digitised (through the Moodle platform and the Studium virtual campus) prior to the onset of the pandemic. This meant that the students participating in this study were used to undertaking learning tasks and knowledge-building via the virtual campus, both with the support of digital technology and without it.

Nonetheless, the results forthcoming from the semi-structured interview indicate that the situation caused by COVID-19 might have prompted the students to be more predisposed to highlight the use of digital technology as a support for knowledge-building. According to Aguilera-Hermida (2020) and Murphy (2020), this is because the lockdown enabled students to become accustomed to the use of digital technology in their learning and knowledge-building processes.

Despite the fact that, as already mentioned, our results coincide with those reported in similar studies conducted pre- and post-pandemic (Coşgun Ögeyik, 2022; DeSchryver, 2017), we understand that the conditions under which this study was held are a good reason for using these instruments in support of future research related to this phenomenon and comparing pre- and post-pandemic findings.

## Conclusion

This study upholds the notion that the ways of building knowledge are changing, and this change may be due to the revolution in digital technologies and their occupation of today’s learning spaces. Consistent with what is happening in other social sectors, these technologies are ushering in a reality of multiple interdependencies, where disorder, ubiquity, and chaos reign.

In order to understand how technology is (re)defining our ways of thinking, learning and building knowledge, we consider that, besides the need for empirical studies for corroborating our findings here, we should perhaps focus on approaches outside the field of education. For example, complexity theory could help to understand this new pedagogical reality, and more specifically, the random and unpredictable behaviour of our thinking skills in the face of the “avalanche” of information in digital scenarios.

Finally, we should like to note that this study’s main limitation involves the selection and size of the sample. Although it is adequate for a qualitative-type study, it is somewhat on the small side for generalising the results.

## Data Availability

The datasets generated and/or analysed during the current study are not publicly available to prevent the individual privacy of the participants from being compromised but they are available from the corresponding author on reasonable request.
